# Dynamics of *Onchocerca volvulus* Microfilarial Densities after Ivermectin Treatment in an Ivermectin-naïve and a Multiply Treated Population from Cameroon

**DOI:** 10.1371/journal.pntd.0002084

**Published:** 2013-02-28

**Authors:** Sébastien D. S. Pion, Hugues C. Nana-Djeunga, Joseph Kamgno, Nicholas Tendongfor, Samuel Wanji, Flobert Njiokou, Roger K. Prichard, Michel Boussinesq

**Affiliations:** 1 UMI 233, Institut de Recherche pour le Développement (IRD) and University of Montpellier 1, Montpellier, France; 2 General Biology Laboratory, Department of Animal Biology and Physiology, Faculty of Science, University of Yaoundé 1, Yaoundé, Cameroon; 3 Faculty of Medicine and Biomedical Sciences, University of Yaoundé 1 and Filariasis & other Tropical Diseases Research Centre, Yaoundé, Cameroon; 4 Research Foundation in Tropical Diseases and the Environment, Buea, Cameroon; 5 Department of Biochemistry and Microbiology, Faculty of Science, University of Buea, Buea, Cameroon; 6 Institute of Parasitology, McGill University, Sainte Anne-de-Bellevue, Québec, Canada; Centers for Disease Control and Prevention, United States of America

## Abstract

**Background/Objective:**

Ivermectin has been the keystone of onchocerciasis control for the last 25 years. Sub-optimal responses to the drug have been reported in Ghanaian communities under long-term treatment. We assessed, in two Cameroonian foci, whether the microfilaricidal and/or embryostatic effects of ivermectin on *Onchocerca volvulus* have been altered after several years of drug pressure.

**Methods:**

We compared the dynamics of *O. volvulus* skin microfilarial densities after ivermectin treatment in two cohorts with contrasting exposure to this drug: one received repeated treatment for 13 years whereas the other had no history of large-scale treatments (referred to as controls). Microfilarial densities were assessed 15, 80 and 180 days after ivermectin in 122 multiply treated and 127 ivermectin-naïve individuals. Comparisons were adjusted for individual factors related to microfilarial density: age and number of nodules.

**Findings:**

Two weeks post ivermectin, microfilarial density dropped equally (98% reduction) in the ivermectin-naïve and multiply treated groups. Between 15 and 180 days post ivermectin, the proportion of individuals with skin microfilariae doubled (from 30.8% to 67.8%) in controls and quadrupled (from 19.8% to 76.9%) in multiply treated individuals but the mean densities remained low in both sites. In fact, between 15 and 80 days, the repopulation rate was significantly higher in the multiply treated individuals than in the controls but no such difference was demonstrated when extending the follow-up to 180 days. The repopulation rate by microfilariae was associated with host factors: negatively with age and positively with the number of nodules.

**Conclusion:**

These observations may indicate that the worms from the multi-treated area recover mf productivity earlier but would be less productive than the worms from the ivermectin-naïve area between 80 and 180 days after ivermectin. Moreover, they do not support the operation of a strong cumulative effect of repeated treatments on the fecundity of female worms as previously described.

## Author Summary

Millions of Africans and thousands of Latin Americans are infected with *Onchocerca volvulus*, the filarial worm responsible for onchocerciasis. Since the mid-1990s, control programs rely on annual or six-monthly community treatments with the only safe drug available, ivermectin. If sustained for another 10–15 years, this strategy could lead to elimination of onchocerciasis. Unfortunately, there have been reports of low response to the drug in Ghanaian communities under long-term treatment. Here, we compared the response of *O. volvulus* to ivermectin between a Cameroonian population repeatedly treated and an ivermectin-naïve population. Skin parasite density was assessed before and 15, 80 and 180 days after treatment. Parasite density dropped equally in the two groups (∼98% reduction) by 15 days, in accordance with the expected effect of ivermectin at this time point. In the multi-treated subjects, the repopulation rate of the skin by microfilariae was higher than in the controls from 15 to 80 days after treatment but the microfilarial levels reached similar levels six months after treatment in the two groups. Thirteen years of large-scale treatments may have selected worms less sensitive to the drug. In addition, those treatments had little if any cumulative effect on skin parasite repopulation after an additional treatment.

## Introduction

In the year 2012, onchocerciasis – or river blindness – is on the verge of being eliminated from some foci in Latin America [1] and, in Africa, evidence of the feasibility of its elimination is emerging [2], [3]. Since the mid-nineties, onchocerciasis control programs have mainly relied on the anthelmintic properties of a single drug, ivermectin. This drug has two main effects on *Onchocerca volvulus*, the parasite responsible for onchocerciasis. The first effect is a killing of the embryonic stage of the parasite, the microfilariae (mf). This so called microfilaricidal effect results in a dramatic decrease of microfilarial density in the skin, starting a few hours following ivermectin intake and reaching its nadir about 1-2 months later [4]. The host immune response is thought to contribute to drug induced microfilarial destruction [5].

The second effect of ivermectin on *O. volvulus* prevents the release of mf from the female worms' uteri and is called the embryostatic effect. The newly produced mf are blocked inside the uteri where they die and degenerate within four weeks post-ivermectin [6]. This effect is temporary and mf start progressively repopulating the skin and other tissues about three months after treatment [4].

Since the embryostatic effect of ivermectin is temporary, treatment has to be repeated to maintain the microfilarial densities as low as possible. By reducing the microfilarial reservoir, ivermectin treatments provide a double benefit: at individual level, they prevent or limit the development of clinical signs associated with onchocerciasis, while at the community level, they reduce the intensity of transmission, and consequently the incidence of infection.

In areas covered by the African Programme for Onchocerciasis Control (APOC), populations receive ivermectin once a year through the community-directed treatment with ivermectin (CDTI) strategy. In 2010, after 15 years of activity, APOC and Non-Governmental Development Organizations have helped the endemic countries to successfully extend ivermectin coverage to a total population of approximately 75.8 million people in 16 countries across sub-Saharan Africa [7]. In most CDTI projects, the therapeutic coverage now exceeds the targeted threshold of 65% of the total population. High coverage ensures significant epidemiological impact of CDTIs and the World Health Organization (WHO) estimates that the loss of nearly one million disability-adjusted life years (DALYs) has already been averted and that the prevalence of infection has decreased by about 73% compared with pre-APOC levels [8].

Shift from control to elimination is now on top of the agenda of APOC management and stakeholders. However, on the fringe of this promising picture, there have been reports of drug underachievement in some communities from Ghana under long-term treatment with ivermectin [9]–[12]. In these communities, a number of individuals, qualified as sub-optimal responders, showed a faster than expected repopulation of the skin by mf. Embryograms of adult female worms [13] performed 90 days after ivermectin treatment revealed a high proportion of worms exhibiting reproductive activity (defined by the presence of developmental stages, i.e. morula, coiled mf or stretched mf, in the uteri). These observations raise the possibility that ivermectin resistance may be emerging, which would manifest itself as a rapid resumption of mf release by the adult female worms, resulting in repopulation of the skin with mf earlier than previously reported [6]. In addition to phenotypic expression of sub‐optimal response, a number of studies have shown genetic changes in *O. volvulus* populations subjected to several years of ivermectin treatment [14]–[21]. Among those genes under ivermectin-driven selection, P-glycoprotein (PGP)and beta-tubulin have been shown to be associated with ivermectin resistance in helminth infection of livestock [22], [23].

To date, a direct association between phenotypic resistance and ivermectin-driven genetic selection has yet to be proven but observations made so far, underscored by the massive drug pressure exerted on *O. volvulus*, warrant active parasitological monitoring of ivermectin efficacy. In the present study, we compared the response profiles of *O. volvulus* to ivermectin between a population repeatedly treated with the drug and an ivermectin-naïve population.

## Methods

### Objectives

The objective of this study, conducted in 2007–2008, was to assess whether the microfilaricidal and/or embryostatic effects of ivermectin on *O. volvulus* have been altered after several years of drug pressure. To this end, we have compared the dynamics of *O. volvulus* skin microfilarial densities after ivermectin treatment in two Cameroonian populations with different histories of exposure to the drug: one has benefitted from repeated treatments for 13 years whereas the other originated from an onchocerciasis endemic area where no large-scale treatment has ever been organized.

### Study areas and selection of participants

A group of ivermectin-naïve patients was recruited from 10 neighbouring communities of the Nkam valley (Bayon, Ekom-Nkam, Mboue, Mpaka, Mbarembeng, Bakem 1, Bakem 2, Lonze, Manjibo and Mounko), a forested area located in the Littoral Region which had neither benefitted from any mass ivermectin treatment at the outset of the study nor vector control. As loiasis was known to be present in this area, *Loa loa* microfilaremia was also assessed for each subject. Participants with more than 30,000 mf/ml of blood, and who were consequently at risk of developing severe adverse reaction to ivermectin, were excluded from the study. Throughout the text, we will refer to this ivermectin-naïve area as the control area.

The group of patients subjected to multiple ivermectin treatments was recruited from 22 communities of the Mbam valley (Babetta, Balamba 1, Balamba 2, Bayomen, Bialanguena, Biamo, Biatsotta, Boalondo, Bombatto, Botatango, Boura 1, Diodaré, Gah-Bapé, Kalong, Kon, Lablé, Lakpang, Ngomo, Ngongol, Nyamanga, Nyamsong and Yébékolo). In these communities, annual large-scale treatments with ivermectin have been conducted since 1994. In addition, these patients had taken part in a clinical trial conducted between 1994 and 1997 aimed at evaluating the macrofilaricidal potential of ivermectin [24]. Depending on their treatment group during the trial, they received 4 to 13 doses of ivermectin over this 4-year period. To date, no vector control has ever been implemented in this area.

The patients eligible for the present study were males aged 25 years and over, presenting with at least two palpable onchocercal nodules, but otherwise in good state of health. As ivermectin had been distributed, for over 10 years, in areas not very far from the Nkam valley, eligible subjects of the control population were asked whether they had been previously treated with ivermectin and those individuals who had either received the drug during the previous five years (2003–2007) or on three or more occasions whatever the dates were excluded from the analyses.

### Sample collection and processing

In both sites, individual skin microfilarial density was assessed before the treatment with ivermectin (D0). Treatment was given under direct observation of investigators. We made the assumption that individuals presenting with no or only one single mf in their skin snips at D0, i.e. at least one year after a previous ivermectin treatment (or at their parasitic equilibrium for untreated subjects), would not exhibit skin mf during the six months following ivermectin treatment and thus decided that only those individuals who had at least two mf in the total of their two skin biopsies on D0 would be re-sampled on D15, D80 and D180. Indeed, it would not have been ethically acceptable to perform additional invasive and painful examinations on individuals with no or only one mf to observe a predictable output. The veracity of this assumption was assessed by examining the response profile of the 10% of the less densely infected subjects of each group.

Skin microfilarial densities were assessed using a slightly modified version of the protocol recommended by the Onchocerciasis Control Programme in West Africa (OCP) [25]. Two skin biopsies (one from each posterior iliac crest) were taken using a 2 mm Holth-type punch and, after 24 hours incubation in saline, all emerged mf were counted under a microscope. Since not all mf present in a skin snip have emerged at 30 minutes, we chose to examine all the skin biopsies at 24 h (rather than at 30 minutes or at 24 h for those negative at 30 minutes as recommended by the OCP) to have a more accurate assessment of microfilarial density. In addition, all skin snips were weighed with 0.1 mg precision, allowing a more accurate assessment of the intensity of infection [26]. Throughout the analysis, the microfilarial densities were expressed in mf per milligram of skin (mf/mg).

All patients also underwent a detailed clinical examination before receiving treatment and the location of all palpable onchocercal nodules was recorded on a standard body chart.

### Ethical clearance and agreement

The study received ethical clearance from the National Ethics Committee of Cameroon and was approved by the Cameroonian Ministry of Public Health. All the participants signed an informed consent form before undergoing the first examination.

### Statistical analyses

The main objective of the analysis was to compare the dynamics of microfilarial densities between two groups: a group of subjects who had been exposed to multiple ivermectin treatments and a control group, composed with individuals living in an ivermectin-naïve area.

In the first instance, we compared, between the two groups, (a) the distribution of microfilarial density for each time of observation, and (b) the distribution of individual variation in microfilarial density from D15 to D80 and from D80 to D180. Those comparisons were performed using the nonparametric Kolmogorov-Smirnov test.

Then, since we previously showed that individual factors such as age and number of palpable nodules could influence the temporal dynamics of skin microfilarial densities after a first dose of ivermectin [27], the comparisons were performed using a multivariate regression model, which allows testing the group-effect while adjusting on individual factors. We tested several models that seemed suitable to handle overdispersed data [28] while accounting for the clustered structure of the dataset (with repeated measures per individual). The dynamics of the microfilarial densities during the D0-D180 period was best described using a zero-inflated negative binomial model with robust variance estimates for each parameter [29]. This model was computed using the *zinb* procedure with the cluster-correlated robust variance estimates available in the Stata software [30], [31]. In this model, the variable of interest was the post-treatment microfilarial density and the regressors comprised host age, the number of palpable onchocercal nodules, the study group (ivermectin-naïve or multiply treated), and two interaction terms: one between the study group and the number of days after ivermectin treatment (0 (i.e. prior to treatment), 15, 80 and 180) and one between the study group and the number of nodules. Inclusion of the former interaction term allowed microfilarial repopulation to vary with time post treatment in a different manner for each population. Inclusion of the latter allowed the effect of number of nodules to vary between the groups, i.e. to test whether the contribution of adult worms to microfilarial density, for a given number of nodules, was different between the groups.

In this model, we chose to set up D15, rather than D0, as the baseline time point, so that the model can conveniently assess the change in microfilarial density due to the microfilaricidal effect of ivermectin (between D0 and D15), and then to its embryostatic effect (from D15 to D80 and from D15 to 180). It is true that repopulation of the skin by mf normally starts to occur progressively by 3 months [4]. However, we hypothesized that sub-optimally responding worms potentially present in the multi-treated group could resume their mf production before 3 months, and that could be indicated by a different dynamics of microfilarial density between D15 and D80.

## Results

### Population description at the outset of the study

In the control area, skin snips and calibrated blood smears were obtained from 194 individuals. Among the 30 subjects who harboured *L. loa* microfilaraemia, three presented with more than 30,000 mf/ml and could not be safely given ivermectin.

Of the 191 subjects eligible for ivermectin treatment in this area, 167 (87.4%) had at least one positive skin snip and 159 (83.2%) of them presented with at least two mf in their two skin snips. Among those 159 individuals, 30 declared having taken ivermectin during the previous five years and another individual declared having taken the drug five times before 2003. These 31 individuals were excluded from subsequent analyses. In addition, one individual showed suspicious post-treatment microfilarial densities which suggested that he had probably not swallowed the ivermectin tablets: 34.1 mf/mg on D0, absent on D15, 69.8 mf/mg on D80 and 30.2 mf/mg on D180. This individual was also excluded from statistical analyses, leading to a total of 127 available subjects for the subsequent analyses in the Nkam valley. The median age and mean number of onchocercal nodules in these 127 subjects were respectively 44 years and 3.4 nodules/person (standard deviation (s.d.) 1.5).

In the Mbam valley (repeatedly treated area), 205 subjects provided skin snips at D0, from whom 138 (67.3%) had at least one positive skin-snip but only 122 (59.5%) presented with a total of at least two mf in the two skin snips. All these 122 individuals received ivermectin and were included in subsequent analyses. The median age and mean number of onchocercal nodules in these 122 subjects were respectively 45 years and 4.5 nodules/person (s.d. 2.4).

### Response profiles to ivermectin treatment

**Figure 1:**
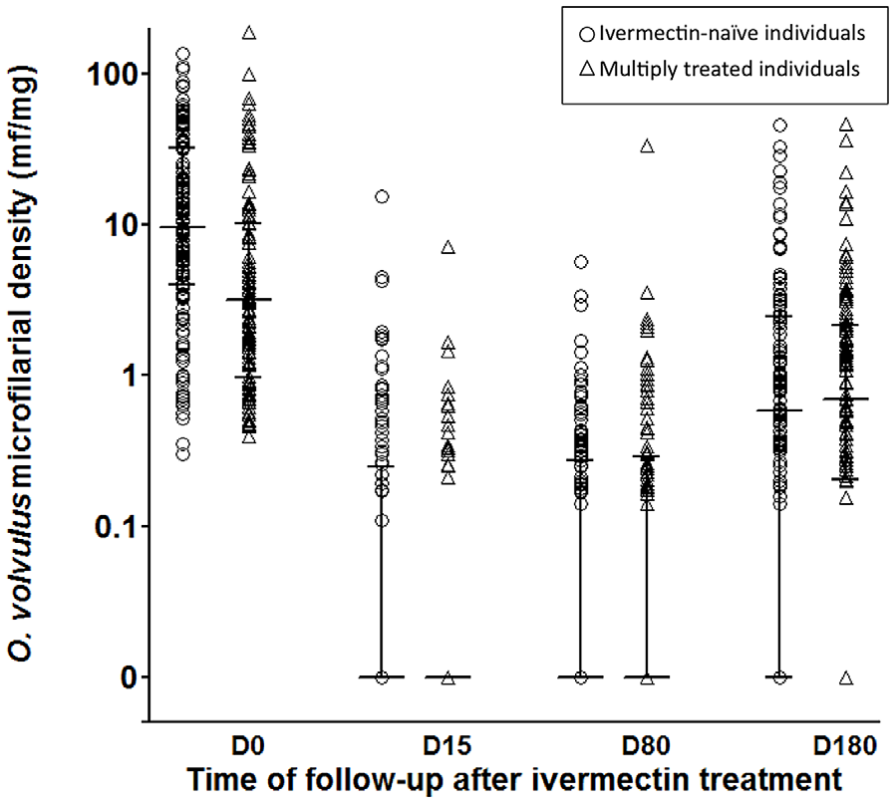
Ivermectin efficacy against *Onchocerca volvulus* in ivermectin-naïve (○) and multiply treated subjects (Δ). Microfilarial density was assessed before and 15, 80 and 180 days after ivermectin in two groups of subjects with different exposure to ivermectin. Bars indicate the median and inter-quartile range of microfilarial density, including zeros.

Microfilarial densities at the different time points of follow-up are shown in Figure 1 and their respective arithmetic means (calculated including zeros) are given in Table 1. As it could be expected, the microfilarial densities recorded just before the 2007 treatment were significantly higher in individuals from the ivermectin naïve area than in those from the multiply treated one (Figure 1, Table 1).

**Table 1:** *Onchocerca volvulus* microfilarial densities (mf/mg) in ivermectin-naïve and multiply treated subjects before and after ivermectin.

	Nkam valley (ivermectin-naïve group)			Mbam valley (repeatedly treated group)			
	No. mf positives/No. examined (%)	Microfilarial density* (s.d.‡)	VMR§	No. mf positives/No. examined (%)	Microfilarial density (s.d.)	VMR	p-value#
Day 0	127/127 (100)	21.8 (24.5)	27.5	122/122 (100)	11.8 (23.9)	48.4	0.001
Day 15	36/117 (30.8)	0.4 (1.5)	5.6	23/116 (19.8)	0.2 (0.7)	2.5	0.429
Day 80	47/117 (40.2)	0.3 (0.7)	1.6	46/112 (41.1)	0.6 (3.2)	17.1	0.999
Day 180	82/121 (67.8)	2.2 (4.6)	9.6	93/121 (76.9)	2.5 (6.1)	14.9	0.366

* arithmetic mean of microfilarial densities (mf/mg), including zero counts.

‡ standard deviation.

§ VMR = variance to mean ratio.

# distributions of microfilarial densities were compared between the two groups using Kolmogorov-Smirnov test.

Two weeks after ivermectin (D15), the proportions of subjects with skin mf dropped to 30.8% and 19.8% in the control and multiply treated groups, respectively, a percent reduction equivalent to ∼70% and 80%. At this time point, the microfilarial densities had dropped in both groups in equal proportions, equivalent to a ∼98% reduction rate of the pre-treatment value (Table 1).

**Figure 2:**
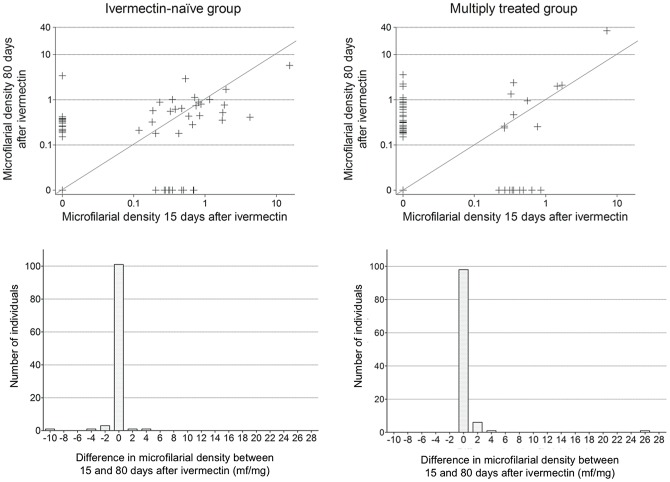
Evolution of *Onchocerca volvulus* microfilarial density (mf/mg) between 15 and 80 days after ivermectin treatment. Microfilarial density at D80 is plotted against density at D15 (scatterplots), for each individual, in ivermectin-naïve and multiply treated subjects. Distribution of individual variation between 15 and 80 days post-treatment is also given (histograms) for the ivermectin-naïve and multiply treated subjects (positive difference means an increase between D15 and D80). Lines in scatterplots represent slope = 1, i.e no change in microfilarial density. X-axis below histograms indicates central values of 2 mf/mg width bins (e.g. 0 indicates microfilarial density between −1 and 1).

Between 15 and 80 days after ivermectin, the proportions of subjects with mf increased by a third to 40.2%, and doubled to 41.1% in the control and multiply treated group, respectively. The mean microfilarial density continued to decrease in the control group but showed an increase in the multiply treated group. However, due to the high variability in microfilarial density, mean values are poorly representative of the panel of actual individual responses and, as shown in Figure 2, from D15 to D80, the subjects showed different profiles of evolution of their microfilarial density. In the multiply treated group, a higher proportion of participants showed an increase in microfilarial density than in the control group (38.7% *vs* 26.7%) (Table 2). At the same time, a higher proportion of subjects showed a decrease in the control group than in the multiply treated group (22.1% *vs* 11.3%) (Table 2). However, comparison of distribution of individual variations from 15 to 80 days post treatment between the two groups did not yield a significant difference (Kolmogorov-Smirnov test, p = 0.171).

**Table 2:** Evolution of *Onchocerca volvulus* microfilarial density after ivermectin treatment in ivermectin-naïve and multiply treated subjects.

	From D0 to D15		From D15 to D80		From D80 to D180	
Percentage of subjects whose microfilarial density:	Ivermectin-naïve	Multiply treated	Ivermectin- naïve	Multiply treated	Ivermectin- naïve	Multiply treated
- increased	0	0	26.7	38.7	66.9	74.8
- decreased	100	100	22.1	11.3	0.9	2.1
- levelled off*	-	-	51.2	50.0	32.2	23.1

* all individuals in this category presented with no detectable skin microfilariae.

**Figure 3:**
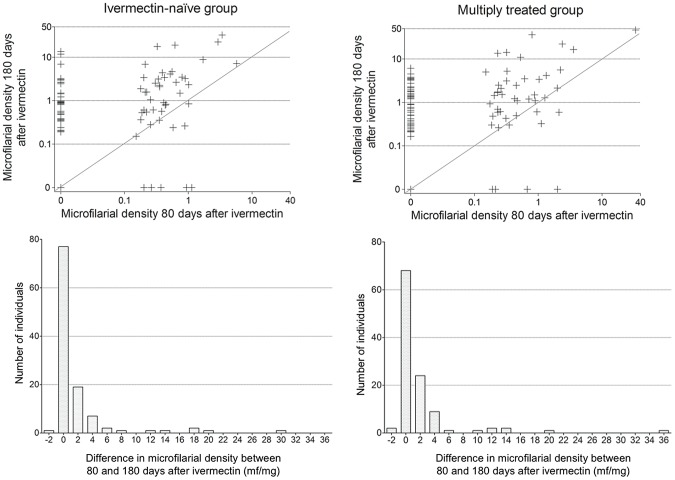
Evolution of *Onchocerca volvulus* microfilarial density (mf/mg) between 80 and 180 days after ivermectin treatment. Microfilarial density at D180 is plotted against density at D80 (scatterplots), for each individual, in ivermectin-naïve and multiply treated subjects. Distribution of individual variation between 80 and 180 days post-treatment is also given (histograms) for the ivermectin-naïve and multiply treated subjects (positive difference means an increase between D80 and D180). Lines in scatterplots represent slope = 1, i.e no change in microfilarial density. X-axis below histograms indicates central values of 2 mf/mg width bins (e.g. 0 indicates microfilarial density between −1 and 1).

From 80 days after ivermectin onwards, reappearance of mf in the skin continued to occur (Table 2, Figure 3). At 180 days post ivermectin, the proportion of subjects with skin mf doubled (increased to 67.8%) in the control group and quadrupled (increased to 76.9%) in the multiply treated one in relation to the values at D15. The mean microfilarial densities remained low in both groups during this time period and reached similar mean values at D180 (2.2 mf/mg in the ivermectin-naïve group *vs* 2.5 mf/mg in the multiply treated group) (Table 1). At D180, only six (5.0%) and seven (5.8%) individuals had microfilarial densities above 10 mf/mg in the control and multiply treated groups, respectively. The distributions of individual variations from 80 to 180 days post-treatment were similar in the two groups (Kolmogorov-Smirnov test, p = 0.345).

In Table 3, we focussed on the microfilarial densities of the 10% less densely infected subjects from each group to assess our assumption that subjects with a very low microfilarial density before treatment would remain mf negative during the six-month follow-up. The tenth percentile of microfilarial density at D0 for the control and multiply treated groups were 1.3 mf/mg and 0.7 mf/mg, respectively. Up to D80, the proportion of subjects showing mf remained low in both sub-groups and those few individuals with a positive skin snip had very low densities. However, at D180, most subjects presented with mf, though still exhibiting moderate microfilarial densities.

**Table 3:** Post-ivermectin microfilarial densities of the 10% less densely infected subjects before treatment.

	D15		D80		D180	
Group	Multiply treated	Ivermectin-naïve	Multiply treated	Ivermectin-naïve	Multiply treated	Ivermectin-naïve
No Examined	13	15	12	15	13	14
No Positives	1	2	2	3	9	8
Mean mf density of positives (mf/mg)	0.2	0.3	0.5	0.3	1.2	1.1

Results of the regression model estimated concurrently on the 4 time points (D0, D15, D80 and D180) are presented in Table 4. They indicate that the decrease in microfilarial density two weeks after ivermectin was similar in the two groups (p = 0.807). Regarding the subsequent period (from D15 to D80), the model indicates a higher increase in microfilarial density in the multiply treated individuals than in the controls (Incidence Rate Ratio (IRR) = 4.045, 95% CI (2.168–7.547), p = 0.001). When considering the longer period D15 to D180, the difference in the increase rate between the two groups was attenuated. The rate tended to be higher in the multiply treated individuals but the difference failed to reach statistical significance (IRR = 2.214, 95% CI (0.835–5.875), p = 0.11). At D180, the distribution of microfilarial densities was similar in the two groups (Kolmogorov-Smirnov test, p = 0.366).

**Table 4:** Zero-inflated negative binomial regression of post ivermectin microfilarial density in ivermectin-naïve and multiply treated subjects.

	Incidence rate-ratio*	[95% Confidence Interval]			P>z
Date of follow-up¶					
Day 0	63.174	31.243	–	127.540	0.001
Day 15	1				
Day 80	0.796	0.479	–	1.322	0.379
Day 180	6.007	2.744	–	13.148	0.001
Study group¶					
Ivermectin-naïve	1				
Multiply treated	0.310	0.111	–	0.860	0.024
Age	0.981	0.967	–	0.995	0.009
Number of palpable nodules	1.186	1.064	–	1.320	0.002
Number of palpable nodules x Study group	1.047	0.835	–	1.312	0.689
Date x Study group					
Day 0 x Multiply treated	1.114	0.467	–	2.656	0.807
Day 15 x Ivermectin-naïve	1				
Day 80 x Multiply treated	4.045	2.168	–	7.547	0.001
Day 180 x Multiply treated	2.214	0.835	–	5.875	0.110
Constant	0.469	0.150	–	1.147	0.194
Zero inflation part					
Number of palpable nodules	−0.415	−0.558	–	−0.273	0.001
Constant	1.701	1.336	–	2.167	
Dispersion parameter (k)	0.618	0.478	–	0.799	

* Results are presented as incidence rate ratios (IRR) for the negative binomial model. For quantitative variables (e.g. age), IRR is the ratio of mean *O. volvulus* microfilarial density when explanatory factor is [x+1] to mean microfilarial density when explanatory factor is [x] (holding all other variables constant). For qualitative variables (e.g. study group) the IRR is the ratio of mean microfilarial density for that category compared to the baseline category.

¶ Baselines for categorical variables are Date of follow-up = D15 and Study group = ivermectin-naïve.

**Figure 4:**
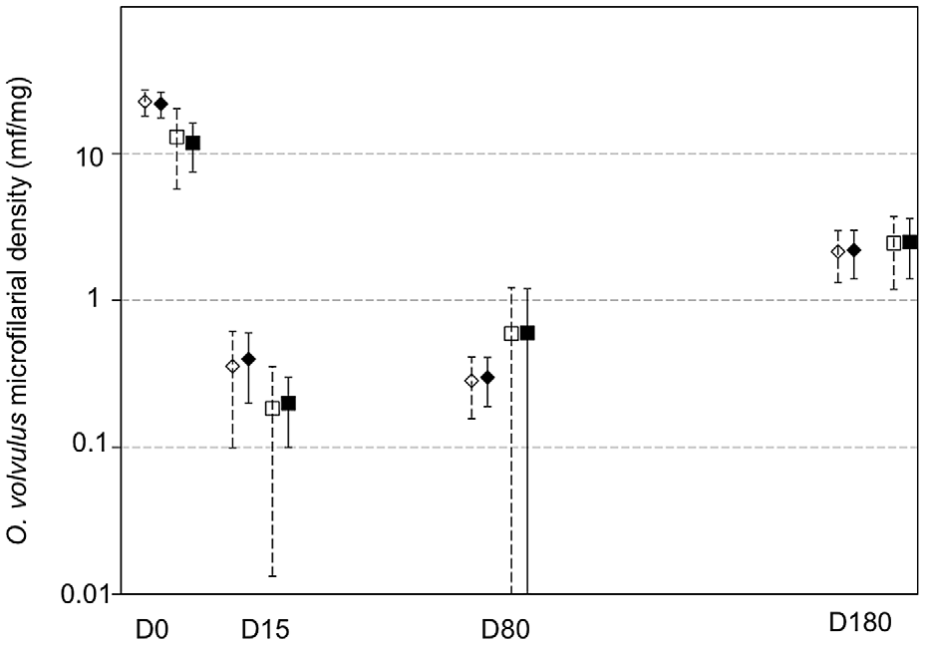
Observed and predicted mean microfilarial density in ivermectin naïve (diamonds) and multiply treated (squares) groups. Filled symbols represent observed means and open symbols represent zero-inflated negative binomial predictions of the mean. Bars indicate 95% confidence intervals for both observations and predictions.

**Figure 5:**
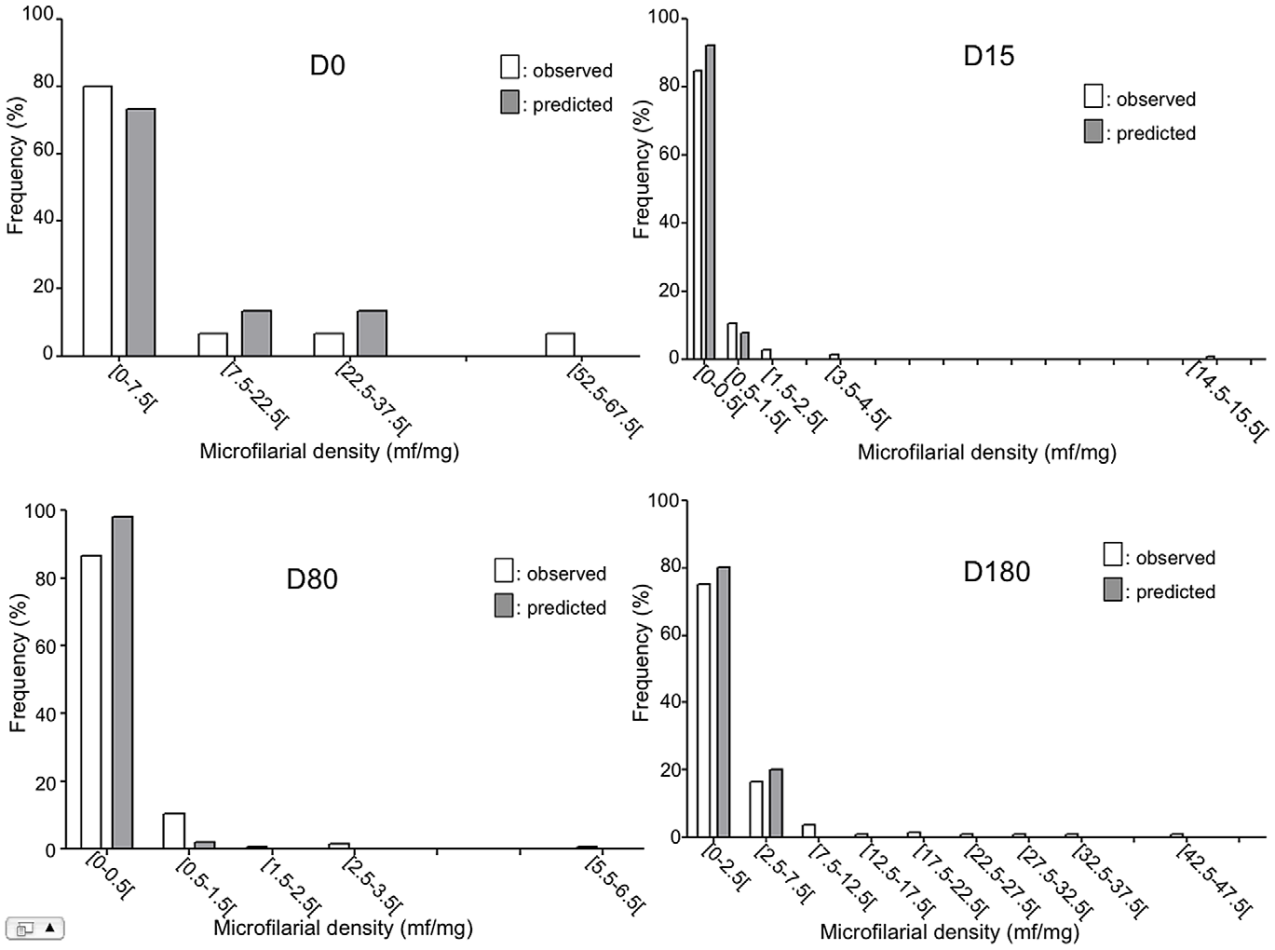
Observed (grey bars) and predicted (empty bars) distributions of microfilarial density in ivermectin-naïve subjects.

**Figure 6:**
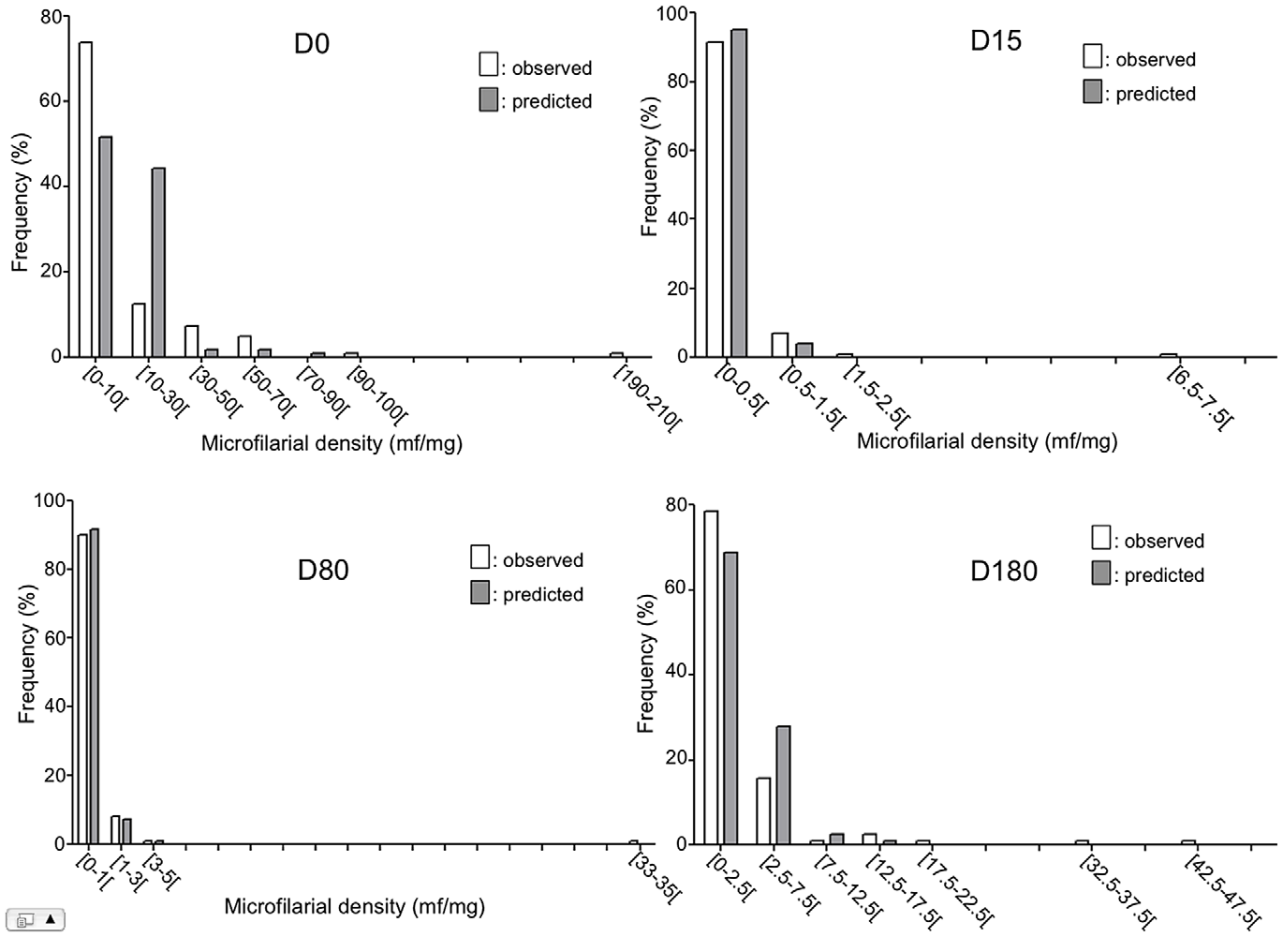
Observed (grey bars) and predicted (empty bars) distributions of microfilarial density in multiply treated subjects.

Beside this, the model shows that age was negatively associated with post-treatment microfilarial densities (p = 0.009), with younger individuals more likely to have higher densities than their older counterparts, and that the number of nodules was positively associated with the post-treatment microfilarial densities (p = 0.002) (Table 4). However, the influence of the number of nodules on microfilarial density was not different between the two study groups (p = 0.689). The mean microfilarial density (and its 95% CI), as predicted by the model for the different times of follow-up, is presented for each of the two study groups in Figure 4. In addition, the frequency distributions of observed and predicted values at the different time points are shown in Figures 5 and 6 for the control group and the multiply treated group, respectively.

## Discussion

To assess whether the effects of ivermectin on *O. volvulus* had been altered after repeated treatments, we aimed at comparing the dynamics of microfilarial densities from 0 to 180 days post-ivermectin between two populations with contrasting histories of treatment (an ivermectin-naïve population and a population exposed to 13 years of treatments) but otherwise similar. Despite our efforts to compose two strictly matched groups (on age, sex and level of infection), the level of infection at day 0 was not even in the two groups, with higher number of nodules and lower mf density in the multiply-treated group than in the control one. The difference in microfilarial density between the two groups at day 0 was expected as a result of previous ivermectin treatments in the Mbam valley. However, the higher number of nodules in the multi-treated participants can result from (a) the fact that the initial level of endemicity of onchocerciasis in the Mbam valley was higher than that in the Nkam valley but also from (b) a possible bias in the inclusion process. Regarding (a), the higher level of endemicity of onchocerciasis in the Mbam valley compared to that in the Nkam valley raises the concern that the worm populations, in particular the age and fecundity of female worms, may differ as a consequence of dissimilar transmission dynamics in the two sites. Indeed, in Liberia, it has been observed that the proportions of old and dead female worms were generally higher in hypo-endemic villages (28% and 18%, respectively) than in hyper-endemic ones (7% and 12%) [32]. We analyzed the population of adult worms collected in the Mbam valley at the outset of the 1994–1998 clinical trial [33]. In this study, the mean number of fecund female worms per nodule was similar in the four groups of villages defined by endemicity level (0.99, 0.90, 0.97 and 0.99 female/nodule in the respective community microfilarial load classes 10–40, 41–60, 61–70 and >70 mf/skin snip). In addition, embryograms of adult worms collected as part of the present study have been analyzed. Preliminary results indicate that, on day 0 (before ivermectin treatment was given in 2007), the female worms' fecundity was similar in the two sites [34]. Further analysis of the worm populations from the two sites would be needed to assess precisely whether they differ significantly or not but the observations made so far suggest that they are not dissimilar. Regarding (b), to be included in the follow-up, participants had to present with, in addition to at least two palpable nodules, at least two mf in their skin snips before ivermectin was given. Amongst the respectively 191 and 205 subjects presenting with at least two palpable nodules in the control and multiply treated group, a significantly higher proportion of participants were excluded from the multiply treated population (40.5%) than from the ivermectin-naïve population (16.8%). This may have resulted in the selection of the most heavily infected subjects (in terms of adult worm load) in the multi-treated area where the participants have had their microfilarial density decreased by previous ivermectin treatments. The rationale for not including individuals with less than two mf in the total of their two skin snips at D0 in the follow-up was to avoid less informative invasive examinations, based on the hypothesis that most subjects, if not all, with a very low microfilarial density before treatment (i.e. at least one year after a previous ivermectin treatment or at their parasitic equilibrium for untreated subjects) would remain mf negative during the six-month follow-up. The response profiles of the less densely infected subjects (the closest similar subjects to those who have not been included) show that this assumption may be acceptable up to D80. However, at D180, significant proportions of subjects had positive skin-snips. If a similar trend occurs in individuals with less than 2 mf/2ss before ivermectin treatment, it may be of relevance to re-examine them at six months or subsequent dates after treatment. To test further whether the exclusion of the subjects had an impact on the comparison between the groups, we also assessed the zero-inflated model on the full sample of subjects examined at D0 (i.e. including the excluded subjects), generating unobserved post-treatment data under two extreme scenarios. Under the first one, we assumed that the microfilarial density of all excluded subjects dropped to zero at D15 and remained at zero at the subsequent time points. Under the second one, we assumed that the microfilarial density of all excluded subjects dropped to zero at D15 and remained at zero at D80 but reached its pre-treatment level at D180. In both cases, the results were very similar to those obtained when the subjects with less than 2 mf/2ss before ivermectin treatment were not included (results not shown), suggesting that their exclusion did not bias the results, in particular the difference of repopulation rate observed between the two groups between D15 and D80.

Nevertheless, to account for this difference in number of nodules, we have assessed the possible effect of exposure to ivermectin (ivermectin-naïve *vs* multiply treated) while adjusting on the number of nodules (as a covariate in the multivariate model). Since the distributions of number of nodules in the two groups of subjects still largely overlap, we think that our study provides robust and reliable results. This being said, were another similar study to be conducted in the future, we would suggest performing a strict matching of individuals on the number of palpable nodules.

Fifteen days after ivermectin treatment, microfilarial densities were expected to be very low [4]. Our observations confirmed that they were indeed very low in both study groups. Despite a higher decrease in the proportion of subjects with mf in the multiply treated group from day 0 to day 15, the longitudinal model indicated that the decrease in microfilarial density was similar in the two groups. As a first observation of this study, the very low levels of skin mf 15 days post-treatment in the multiply treated group do not indicate any alteration in the microfilaricidal effect of ivermectin after 13 years of large-scale use in the area. These results confirm those observed in Ghana [11], and that the percent reductions in microfilarial load are also similar to those expected in a normally responding parasite population [4].

Between two weeks and 80 days post-ivermectin treatment, most subjects of both groups did not show detectable skin mf, with even proportions in the control and multi-treated groups (51.1% and 50%, respectively), yet microfilarial repopulation of the skin occurred in a minority of subjects (26.7% and 38.7% in the control and multi-treated groups, respectively). Results of multivariate analysis conducted on this time period (D15–D80) indicate that, on average, the rate of skin repopulation was higher in the multi-treated group than in the control group (IRR ∼4). This trend is, in part, due to the higher proportion of subjects whose microfilarial load increased between D15 and D80 in the multi-treated group. This may indicate that the embryostatic effect of ivermectin has been relaxed more rapidly and/or in a greater number of worms in subjects from the multi-treated areas than in those from the ivermectin-naïve zone.

When extending the follow-up from 15 days to six months after ivermectin, the difference between the groups was attenuated and did not reach statistical significance (IRR ∼2, p = 0.11). At six months post-treatment, the distribution of microfilarial density was similar in the two groups. This may indicate that, after ivermectin, the worms from the multiply treated group may recover mf productivity earlier (more worms resume mf productivity between D15 and D80 in the multiply treated group) than the worms from the naïve one but, from D80 to D180, the former worms would be less productive than the latter. This picture is in accordance with previous findings, in which repeated ivermectin treatments have been found to cause a genetic selection of *O. volvulus* associated with a lower reproductive rate in female worms [18]. It would therefore be of utmost interest to perform genetic analysis of the worms collected as part of the present study to assess whether this difference is genetically driven.

The patients included in the multi-treated group were given annual or 3-monthly ivermectin treatments (thus 4 or 13 doses) between 1994 and 1997 as part of a carefully monitored clinical trial. Subsequently, during 9 years, they were offered ivermectin once a year as part of CDTI campaigns. Thus, before the treatment given as part of this study (in 2007), they had received between 4 and 22 ivermectin doses over a 13-year period. We did not manage to document precisely the history of treatment for the period 1998–2006 for each participant in the multiply treated group. Attempts of retrospective interviews showed that this approach suffers from serious recall bias and official registers used by the community distributors during CDTI campaigns were not available or not fully reliable. Nevertheless, we can assume that a significant proportion of adult worms present in 2007 in the multiply treated hosts had been subjected to several rounds of ivermectin. As indicated by the model (interaction term between the number of nodules and the study group), the contribution of adult worms to microfilarial density, for a given number of nodules, was not different between the groups. Therefore, observing similar levels of microfilarial density during six months after administration of a dose of ivermectin in the two groups does not suggest that, in those female worms which have been repeatedly targeted by ivermectin, fecundity has been cumulatively and irreversibly reduced, as it has been proposed in the past [35]. Thus, together with recent findings obtained by Bayesian modelling of the effect of six-monthly ivermectin treatments on skin microfilarial density [36], our observations do not support the operation of a strong cumulative effect of repeated treatments on the fecundity of female worms. Yet, our present analyses are strictly based on assessment of mf reappearance in the skin as an indicator of ivermectin efficacy. Microfilarial density represents the production of a pool of worms living in the host. Results presented by Osei-Atweneboana and others [12] suggest that worms contribute unequally to skin repopulation, possibly those less sensitive to ivermectin contributing the most to the re-increase in skin density after multiple treatments. The possibility that the same pattern occurs in the patients from the Mbam valley cannot be discarded based on the present results. Further analysis of the worms' reproductive status, in particular in those subjects harbouring the highest microfilarial densities 180 days after ivermectin, may give a more comprehensive picture of how the worms have been affected by ivermectin pressure.

One important finding is the demonstration that the microfilarial density after treatment is significantly associated with the two individual level factors included as covariates in the model, namely age and number of nodules. This holds true both after a first treatment [27], and after repeated doses of ivermectin (present data in multiply treated group). Specifically, the number of nodules seems to have a strong effect on the post-ivermectin microfilarial density, with any increase in one nodule associated with a 14.9% increase (conditionally on average values of other covariates) in microfilarial density during the period of follow-up (D15–D180). During a previous study conducted in the Mbam area, we observed that the number of worms per nodule was fairly constant and independent of the number of palpable nodules [33]. Therefore, we can assume that the number of palpable nodules is positively correlated with the number of adult worms actually present in the host. If this assumption is correct, it is not surprising to observe that the more nodules in the host, the higher the rate of skin repopulation by mf after ivermectin treatment. This assumption may not be true in all onchocerciasis foci [37] but, should it apply in endemic communities of central Ghana, it may explain, at least partly, the observations of sub-optimal responses that brought up some controversy regarding the development of ivermectin resistance [11], [38]–[42]. In the Ghanaian study, a particularly noteworthy figure is that those communities showing statistically [28] higher than expected rates of skin repopulation had higher nodule prevalences (16.1%, 19.3% and 22.9%) than communities responding as expected (4%, 5.1%, 5.6%, 6.0%, 6.9% and 15.4%). Since nodule prevalence is probably correlated with the number of adult parasites in the population, the higher rates of skin repopulation in the four suboptimally responding Ghanaian communities may be partly due to differences in intensity of infection, as assessed by nodule prevalence at the time of the study. Adjusting the individual responses on individual nodule density may be critical in the assessment and monitoring of ivermectin efficacy in onchocerciasis. Nonetheless, because the relationship between the number of nodules and the repopulation rate may not be linear, inferring this correlation to indicators relevant at the community level (nodule prevalence and mean percentage of microfilarial density reduction) certainly requires further statistical analysis and we would encourage such modelling studies to be conducted.

One of the most striking observations made during the study was the high number of nodules harboured by the subjects from the Mbam valley. As a decrease in the prevalence of nodules following mass chemotherapy has been documented in several areas [43], [44], such a situation was quite unexpected after 13 years of repeated ivermectin treatments, including nine years of CDTI in the whole region. Although it was out of the scope of this study, we would advocate for assessment of the impact of CDTI in the area. Persistence of infection after almost a decade of large-scale treatments may be related to sub-optimal response of the worms to ivermectin but also to unsatisfactory therapeutic coverage, or both.

We think that the significant association between the number of palpable nodules and post-ivermectin microfilarial dynamics is an important finding. It has been argued that the diagnostic value of nodule palpation tends to be poor in communities with low or moderate endemicity [45] but the results of the present study show that the distribution of nodules may have to be considered when evaluating the impact of ivermectin treatments on the levels of endemicity of onchocerciasis in those areas that were originally hyperendemic. Still, we have to keep in mind that for this study, inclusion criteria required that participants were males presenting with at least two palpable nodules and the influence of nodules on the microfilarial density after treatment should be also assessed in other strata of the population. Further analyses of existing data including a more representative sample of the general population (e.g. community trials of ivermectin during which microfilarial density and palpable nodules would be assessed) should be encouraged.

Recently, elimination of onchocerciasis using ivermectin alone has been achieved in some African foci [2], [3]. Yet, it is thought to be feasible only under specific conditions such as both a low to moderate initial level of endemicity and a high, sustained, therapeutic coverage. In most parts of Africa, similarly to the situation in the Mbam valley, annual treatment may not be effective in efforts to eliminate onchocerciasis. A failed approach would lead to repeat the treatment virtually indefinitely, and may well contribute to increased risk of drug resistance. To achieve the goal of transmission interruption, programme managers should consider the possibility to provide more frequent rounds of ivermectin treatments, as well as developing new means to ensure that maximal therapeutic coverage is reached every year. Beside, vector control should be considered, in addition to large scale chemotherapy, in areas where the annual transmission potential is particularly high [46].

Lastly, some host-related genetic factors may also be responsible for a fraction of the variability in the response to infection following ivermectin treatment. Genetic factors driving different immune response against *O. volvulus* in the natural course of infection have been identified [47], [48] but their potential role in ivermectin efficacy against this parasite has still to be assessed.

## Supporting Information

Text S1 This file contains the list of abbreviations used throughout the manuscript.
(DOCX)
