# Supplementary material for: Dynamics of Onchocerca volvulus Microfilarial Densities after Ivermectin Treatment in an Ivermectin-naïve and a Multiply Treated Population from Cameroon
Source: PLoS Negl Trop Dis. 2013 Feb 28;7(2):e2084. doi: 10.1371/journal.pntd.0002084 (PMC3585010; doi:10.1371/journal.pntd.0002084)
Supplement: Text S1 — This file contains the list of abbreviations used throughout the manuscript. (DOCX) [file pntd.0002084.s001.docx]

**List of abbreviations**

APOC: African Programme for Onchocerciasis Control

CDTI: Community Directed Treatment with Ivermectin

DALY: Disability Adjusted Life Years

IRR: Incidence Rate Ratio

mf: microfilariae

OCP: Onchocerciasis Control Programme in West Africa

PGP: P-glycoprotein

s.d.: Standard Deviation

VMR: Variance to Mean Ratio

WHO: World Health Organization
